# Prevalence and determinants of occupational Injuries among welders in small scale metal workshops in Wakiso District, Uganda

**DOI:** 10.24248/eahrj.v5i1.657

**Published:** 2021-06-11

**Authors:** Brian Itiakorit, Esther Bayiga Zziwa, Jimmy Osuret

**Affiliations:** a College of Health Sciences, School of Public Health, Makerere University, Kampala_Uganda

## Abstract

**Background:**

Injuries are a public health concern accounting for 2.78 million fatalities globally. Welders are exposec to a broad range of injuries (e.g. cuts, burns, eye injuries, skin irritations, and musculoskeletal disorders) and yet, there is paucity of information on context specific determinants to inform injury prevention and control. This study determined the factors associated with occupational injuries among welders in Uganda.

**Methods:**

A cross-sectional study was conducted among welders in Wakiso District, Uganda. Pretested and translated questionnaires were used to collect data from 327 randomly selected respondents using face to face interviews. 2 parishes were purposively selected, and 20 metal workshops were systematically selected in each parish. Descriptive statistics and adjusted odds ratios were computed

**Results:**

A high prevalence 287 (87.8%) of self-reported occupational injuries was found among welding workers with cuts/burns 242 (84.3%) and eye injuries 180 (62.7%) reported as the most sustained injuries. Occupational injuries were associated with being a causal labourer with informal training (AOR 4.70 (2.03–10.84)) and working for longer hours (AOR 2.63 (1.26–5.51)). Those with more work experience were less likely to be involved in occupational injuries (AOR 0.30 (0.11–0.84)).

**Conclusions:**

The prevalence of occupational injuries among small-scale welding workers was high and this was associated with learning their trade at work and working for longer hours. Mitigation measures that focus on safety at workplace, advocating for capacity training, and enforcement of workplace regulations should be instituted.

## BACKGROUND

Occupational injuries are a global public health concern with more than 2.78 million fatalities and approximately 374 million non-fatal occupational injuries sustained annually at workplaces.^1^ In the recent years, Low and Middle-Income Countries (LMICs) have accounted for three quarters of the global burden of fatal occupational injuries which is not the case in developed countries that have seen a steady decline.^2^ In LMICs, standards and practices are below acceptable levels due to the laxity in the enforcement of occupational health and safety regulations.^3^ Across the African region, welders continue to content with the effects of occupational injuries and yet there is inadequate literature on the injury burden. In Uganda, Micro, Small, Medium Enterprises (MSMEs) Enterprises play a vital role in creating employment and contributes 20% to the country's Gross Domestic Product (GDP).^4^ However, occupational hazards and injuries continue to be a burden especially among people working in Small Scale Metal Workshops (SSMW).^5^

People who work in SSMW indulge in high exposure activities such as heavy lifting, cutting, polishing and joining of metal pieces through gas and/or metal-arc welding. Such activities expose the workers to both safety and health hazards such as fire, noise, electric shock, tips and falls, glare and ergonomic hazards.^6–8^ The high prevalence of occupational injuries among the welding workers is usually attributed to individual and workplace factors such as age, lack of institutional training, work experience, long working hours, failure to implement safety regulations, alcohol/tobacco use and use of sub standardised Personal Protective Equipment (PPE).

Uganda has a legal framework under the Occupational Safety and Health (OSH) Act 2006 that provides the rights, duties and responsibilities for workers to ensure work place safety.^9^ However, progress in reducing the incidence of occupational injuries remains sub optimal since the compliance to OSH requirements has been documented to be low among welders and it has been attributed work environment and negligence of welders.^10,11^ Most studies conducted in the Ugandan context have largely focused on knowledge, attitudes and practices towards utilisation of safety and health practices among welders^12,13^ with limited information on the factors. Estimating the prevalence of occupational injuries and associated factors among welders is important in generating information that will guide injury prevention and control programs among welders. The purpose of this study was to estimate the prevalence of occupational injuries and associated factors among welders in Uganda.

## METHODS

### Study Design

A descriptive cross-sectional study that utilised quantitative data collection methods was conducted between October 2018 and January 2019. This study was conducted among welders working in SSMW within Kajjansi town council, Wakiso district.

### Study Setting

The study was conducted in Kajjansi town council, a peri-urban area found in Wakiso district, Uganda. Wakiso District is located in the central southern part of the Central Region of Uganda and has an estimated population of 1,997,418.^14^ Kajjansi town council is situated in Ssisa Sub County approximately 16 kilo metres South of Kampala, the capital of Uganda. Kajjansi town council is made up of 12 parishes which are both rural and urban. Majority of the population are employed in informal businesses such as hairdressing, carpentry, tailoring, welding among others. The numerous informal welding workshops found in Kajjansi town council employ a significant population within the town council.

### Sample Size Estimation

The study sample size (n) was calculated using the Leslie Kish formula (1964) at 95% Confidence Interval (Z), a Prevalence (P) of occupational injuries among welders working in SSMW was assumed at 50%, Marginal Error (e) of 5.5% and a 10% non-response rate was usedd.


$$\text{n}=\frac{Z^{2}P(100\%-P)}{\varepsilon^{2}}$$

Z = 1.96 (standard normal deviate at 95% confidence interval)


$$\text{n}=\frac{1.96^{2}50\%(100\%-50\%)}{5.5{\%}^{2}}=317.5\approx 318$$

Adjusting for non-response


$$\begin{array}{ll} \text{n} & =318+318\times 10\%\\ & =349 \end{array}$$

Therefore, the study sample size was 349 respondents. However, we collected information from 327 respondents.

### Sampling and Data Collection Sampling Procedure

Two (2) parishes in Kajjansi town council i.e. Namulanda and Kitende were purposively selected for the study. 20 metal workshops were systematically selected from each parish. Respondents from each workshop were then randomly sampled and a number proportionate to the existing number of people was selected. A maximum number of 10 respondents per workshop were selected randomly. The inclusion criteria included: being at least aged 18 years, currently working at SSMW for at least 6 months and being present at the time of the study. Exclusion criteria included being absent at the time of the study, refusal to consent and not being in a good mental health i.e. being under the influence of alcohol or illicit drugs.

### Data Collection

A questionnaire with both open-ended and closed-ended questions was used. It included sections on; socio-demographic data, job description, PPE ownership and utilisation, workplace condition and morbidity of injuries. The questionnaires were developed in English then translated to the local language, Luganda. Pretesting of the questionnaires was done to ascertain reliability, validity and quality of the study. 2 research assistants were trained on the data collection techniques and were briefed about the study objectives. Informed consent was sought from eligible respondents and the questionnaire was administered in Luganda at a place of convenience and privacy to the participant. Data was collected through face to face interviews and interviews took an average of 15 minutes per participant. Occupational injuries were self-reported as respondents were asked about injury history within the past 1 year prior to the study.

### Data Management and Analysis

Data collected was checked daily for any inconsistencies and incompleteness. Data was entered using EpiData 3.02 software, later cleaned and analysed using STATA 14.0 (StataCorp, Texas, USA) analytical software. Descriptive, Bivariate and Multivariate data analyses were done. Frequencies and percentages were used to describe the variables while Crude Odds Ratios (COR) and Adjusted Odds Ratios (AOR) at 95% confidence intervals and p-values of ≤0.05 were presented in tables and later interpreted.

### Ethical Approval and Consent to Participate

This study was approved by the Makerere University School of Public Health Research and Ethics committee. Permission to conduct this study was sought from Kajjansi Town council, Wakiso district and informed consent was sought from all the study respondents.

## RESULTS

### Socio Demographic Characteristics

The questionnaire completion rate was at 327(93.7%). All respondents were males with a mean age of 25 years (SD±8.08). Most respondents, 206(63.0%) were below 25 years and 147(45.0%) had attained primary level education. More than half of the respondents, 209 (63.9%) had worked for less than 5 years.

### Workplace Characteristics

Almost all of the respondents, 316 (96.6%) indulged in cutting of metal pieces. Majority of the respondents 205 (62.7%) were casual labourers who got the skill through on the job learning and more than half, 247(75.5%) worked for 8 hours and above per day. More than three quarters of the respondents 301(92.0%) had work targets and more than half of the respondents 209 (63.9%) had below 5 years of work experience (Table 1).

**TABLE 1:** Workplace Characteristics

Variable	Frequency (n=327)	Percentage (%)
Roles at the workshop^*^		
Heavy lifting	145	44.3
Cutting	316	96.6
Welding	295	90.2
Painting	167	51.1
**Training attained**		
Institutional training	122	37.3
Causal labourer (No formal training)	205	62.7
**Work hours per day**		
Below 8 hours	68	20.8
8 hours and above	247	75.5
**Do have work targets**		
Yes	301	92.0
No	26	8.0
**Work experience (years)**		
Below 5 years	209	63.9
5 years	23	7.0
Above 5 years	95	29.1

* -Multiple response

### Prevalence of Occupational Injuries

The overall prevalence of occupational injuries among with elders was 287 (87.8%) and majority of which 242 (84.3%) had sustained burns/cuts. Almost all of the respondents, 317 (96.9%) owned PPE and 312 (98.4%) utilised their PPE during work. Most of the respondents, 287(90.5%) used goggles during their work (Table 2).

**TABLE 2:** Occupational Injuries Sustained in the Past One Year among Metal Workshop Workers

Variables	Frequency (n=327)	Percentage (%)
**Sustained any occupational injuries?**		
Yes	287	87.8
No	40	12.2
Injuries sustained^*^	**n=287**	
Burns and cuts	242	84.3
Eye strain	180	62.73
Head and back injuries	99	34.5
Falls and trips	57	19.8
Fractures	84	29.3
**PPE ownership**		
Yes	317	96.9
No	10	3.1
**PPE utilisation**	**n=317**	
Yes	312	98.4
No	5	1.6
Type of PPE used^*^	**n=317**	
Goggles	287	90.5
Face masks	110	34.7
Gloves	241	76.0
Overall and boots	254	80.1

* -Multiple response

### Factors Associated Occupational Injuries among Welders

At bivariate analysis; those aged above 35 years (COR=0.38, CI=0.16–0.85, *P=0.019*), those who had worked for more than 5 years (COR=0.35, CI=0.17–0.72, *P=.004*) and those who had no work targets (COR=0.27, CI=0.11–0.66, *P=.005*) were less likely to have sustained occupational injuries. Respondents who worked as causal labourers (COR=3.69, Cl=1.85–7.40, *P<.001),* worked for 8 hours and above (COR=2.58, CI=1.31–5.10, *P=.006*) and consume alcohol (COR=2.73, CI=1.25–5.95, *P=.011*) were more likely to have sustained occupational injuries (Table 3).

**TABLE 3:** Factors Associated with Occupational Injuries among Welders

Variable	Occupational Injuries				Crude Odds Ratio (COR) at 95% Confidence Interval (C.I)	*P-value*
	Yes		No			
	F	%	F	%		
**Age groups (years)**						
Below 25	184	64.1	22	55.0		
26–35	68	23.7	7	17.5	1.16 (0.47–2.84)	.743
Above 35	35	12.2	11	27.5	0.38 (0.16–0.85)	.019^*^
**Education level**						
Never went to school	29	10.1	4	10.0		
Primary	134	46.7	13	32.5	1.42 (0.43–4.68)	.562
Secondary/tertiary	124	43.2	23	57.5	0.74 (0.24–2.31)	.609
**Marital status**						
Single/divorced	190	66.2	29	72.5		
Married	97	33.8	11	27.5	1.35 (0.64–2.81)	.429
**Work experience (years)**						
Less than 5 years	192	66.9	17	42.5		
5 years	19	6.6	4	10.0	0.42 (0.13–1.38)	.153
Above 5 years	76	26.5	19	47.5	0.35 (0.17–0.72)	.004^*^
**Training attained**						
Institutional training	96	33.4	26	65.0		
Causal labourer (No formal training)	191	66.6	14	35.0	3.69 (1.85–7.40)	**<.001**
**Work hours**						
Below 8 hours	69	24.0	18	45.0		
8 hours and above	218	76.0	22	55.0	2.58 (1.31–5.10)	.006^*^
**Have work target?**						
Yes	269	93.7	32	80.0		
No	18	6.3	8	20.0	0.27 (0.11–0.66)	.005^*^
**Tobacco use**						
Yes	17	2.9	1	2.5		
No	270	94.1	39	97.5	0.41 (0.05–3.15)	.389
**Alcohol use**						
Yes	35	12.2	11	27.5		
No	252	87.8	29	72.5	2.73 (1.25–5.95)	
						.011^*^

* -Statistically significant at P<.05

At multivariate analysis after controlling for potential confounders, work experience of more than 5 years (AOR=0.30, CI=0.11–0.84, *P=.021*) and working without targets (AOR=0.20, CI=0.07–0.57, *P-value=.002*) were protective while working as a casual labourer (AOR=4.70, CI=2.30–10.84, *P<.001*) was significantly associated with occupational injuries (Table 4).

**TABLE 4:** Factors Influencing Occupational Injuries among Small-Scale Metal Workshop Workers

Independent variable	Crude Odds Ratio (COR) at 95% Confidence Interval (C.I)	Adjusted Odds Ratios (AOR) at 95% Confidence Interval (CI)	*P-value*
**Age**	0.97 (0.93–1.01)	1.04 (0.98–1.11)	.153
**Work experience (years)**			
Less than 5 years			
5 years	1.16 (0.47–2.84)	0.35 (0.10-1.27)	.112
Above 5 years	0.38 (0.16–0.85)	0.30 (0.11–0.84)	.021^*^
**Training attained**			
Institutional training			
Causal labourer (No formal training)	3.69 (1.85–7.40)	4.70 (2.03–10.84)	<.001^*^
**Work hours**			
Below 8 hours			
8 hours and above	2.58 (1.31–5.10)	2.63 (1.26–5.51)	.010^*^
**Have work target?**			
Yes			
No	0.27 (0.11–0.66)	0.20 (0.07–0.57)	.002^*^

* -Statistically significant at P<.05

## DISCUSSION

Majority of the respondents were below 25 years of age and had attained primary level education. Uganda's demographic profile indicates that majority of the population are predominantly the youth who are employed in the informal sector.^15^ This finding is consistent with those found in similar studies conducted in Nepal and India where welders were predominantly male under the age of 25 years.^16,19^

The overall prevalence of occupational injuries among welders working in small scale metal workshops was high at 87.8%. These findings are consistent with those found by other similar studies conducted in South India, Nigeria and Kenya.^16,20,21^ This high prevalence of occupational injuries is due to inadequate enforcement of OSH legislation, inadequate professional job training and inadequate awareness OSH issues related to welding.^12,22^

Burns and cuts on the hands and fingers were the most prevalent occupational injuries sustained and these findings are consistent to those found in Eastern Uganda, Nigeria, Iran and coastal South India.^12,20,23,24^ A review of literature showed that most welders are highly aware of eye protective devices since eye injuries are perceived to be more severe than other injuries.^20,25,26^ Goggles were the commonest PPE used, these findings are consistent with those found in India and Ethiopia.^16,19,27^ This could be attributed to the high awareness about the instant health implications of welding flash light that arise during metal-arc/gas welding and low perception of occupational risk to other body parts.^28–32^ This finding implies that most of the welding workers prioritise eye safety to other body parts.

The finding that causal labourers with no formal training were approximately four times more likely to have sustained occupational injuries as compared to their counterparts indicates that the unprofessional on-the-job training provided is skills-based and little attention is given to safety.^33,34^ Similar findings were found in studies conducted in Nigeria, Nepal and Pakistan.^17,34,35^

This study found out that those welding workers who did not have work targets to be less likely to have sustained occupational injuries and this could be attributed to sufficient time allocated to accomplish a task. A review of literature showed that 8 hours per 5 days per week is maximum exposure limit recommended to welding workers.^36^ Workers with work targets tend to work beyond 8 hours or more than 5 days per week thus becoming more susceptible to making errors that make them sustain occupational injuries.^33,37^

In this study, it was established that workers with more than 5 years of work experience were less likely to have sustained occupational injuries. These findings are similar to those found in studies conducted in Northern Nigeria and southern India^11,38^ and this has been attributed to the appropriate use of PPE as a result of previous exposure to injuries and hazard awareness amongst the experienced welding workers.^27,39^ Supportive supervision should be provided to inexperienced workers in order to ensure guidance and knowledge sharing on issues related to occupational safety.

**Limitation:** The study was cross sectional in design and therefore causality was not possible to assess. The results could have been affected by recall bias as the respondents were required to remember their experience. Nevertheless, the study gives an insight on the factors associated with occupational injuries among welding workers in a peri-urban setting. This information is important for designing appropriate strategies and interventions to curtail occupational injuries through implementation of workplace regulation.

## CONCLUSION

The prevalence of occupational injuries among small-scale welding workers was high and this was associated to the unprofessional on-the-job learning and working for more than 8 hours. These findings call for advocacy for integrating occupational safety and health aspects in the on-the-job trainings amongst welding workers. Existing workplace regulations should be upheld by law enforcement officers and Public health department to ensure employers are not overworked at their workplaces.
